# Childhood Allergy Disease, Early Diagnosis, and the Potential of Salivary Protein Biomarkers

**DOI:** 10.1155/2021/9198249

**Published:** 2021-10-08

**Authors:** N. H. M. Zainal, R. Abas, S. F. Mohamad Asri

**Affiliations:** Department of Human Anatomy, Faculty of Medicine and Health Sciences, Universiti Putra Malaysia, 43400 UPM Serdang, Selangor, Malaysia

## Abstract

Allergic disease has risen to epidemic proportions since the last decade and is among the most common noncommunicable, chronic diseases in children and adolescents worldwide. Allergic disease usually occurs in early life; thus, early biomarkers of allergic susceptibility are required for preventive measures to high-risk infants which enable early interventions to decrease allergic severity. However, to date, there is no reliable general or specific allergy phenotype detection method that is easy and noninvasive for children. Most reported allergic phenotype detection methods are invasive, such as the skin prick test (SPT), oral food challenge (OFC), and blood test, and many involve not readily accessible biological samples, such as cord blood (CB), maternal blood, or newborn vernix. Saliva is a biological sample that has great potential as a biomarker measurement as it consists of an abundance of biomarkers, such as genetic material and proteins. It is easily accessible, noninvasive, collected via a painless procedure, and an easy bedside screening for real-time measurement of the ongoing human physiological system. All these advantages emphasise saliva as a very promising diagnostic candidate for the detection and monitoring of disease biomarkers, especially in children. Furthermore, protein biomarkers have the advantages as modifiable influencing factors rather than genetic and epigenetic factors that are mostly nonmodifiable factors for allergic disease susceptibility in childhood. Saliva has great potential to replace serum as a biological fluid biomarker in diagnosing clinical allergy. However, to date, saliva is not considered as an established medically acceptable biomarker. This review considers whether the saliva could be suitable biological samples for early detection of allergic risk. Such tools may be used as justification for targeted interventions in early childhood for disease prevention and assisting in reducing morbidity and mortality caused by childhood allergy.

## 1. Introduction

### 1.1. The Allergy Epidemic

Allergy is a hypersensitivity reaction triggered by immune system mechanisms. Atopy is defined as a personal or familial tendency to develop IgE antibodies in response to low-dose common environmental antigens (allergens), such as pollens, dust, and various food kinds, as confirmed by a positive skin prick test (SPT). These allergens are environmental factors in which the immune system generally does not develop an immunological response [1, 2]. Allergic sensitization normally starts in childhood, and the number of allergens to which a patient is sensitized might grow with time [3]. Allergic diseases include asthma, atopic dermatitis (AD), allergic rhinitis (AR), food allergies (FA), and anaphylaxis.

Allergic disease has risen to epidemic proportions since the last decade [4–7]. Recent data suggest that prenatal events, such as environmental influences on placental function and fetal programming, have a critical role in determining disease susceptibility. Furthermore, evidence suggests that allergic disease is associated with immune system deviations that occur *in utero* [8, 9]. Th1-dependent antimicrobial immunity suppression in the neonatal period is a consequence of fetomaternal tolerance to prevent fetus rejection, leading to Th2 dominance and, in combination with other events and exposures, could predispose children to allergic disease [10, 11].

Most concerning is the increase in allergic and autoimmune diseases in the last 50 years indicating the susceptibility of immune pathways to modern environmental influences. Allergic diseases affect more than 20% of the population worldwide, especially in industrialised countries [5]. Furthermore, allergic diseases are among the most common chronic noncommunicable diseases in children and adolescents worldwide, with an estimated 50% of all schoolchildren suffering from allergic diseases [12]. Thus, allergy is a complex and heterogeneous disease that presents a significant burden to human health and preventative measures to reduce this burden are urgently required. Early detection of allergic susceptibility may be an approach by which prevention or interventions could be introduced to decrease allergic severity.

In children, asthma frequently coexists with allergies and other allergies which includes AD, AR, and FA [13, 14]. Most young children with severe AD have an increased risk of developing asthma and rhinoconjunctivitis. However, most cases of AD are mild to moderate in the general population. The “atopic march” is a pattern of progression through multiple allergy illnesses in early childhood in which individuals who first present with AD later develop AR and eventually atopic asthma [15]. Saunes et al. investigated the risk of the current asthma and the coexistence of allergy-related diseases in children aged six [16]. According to their findings, although most cases of AD in the general population were mild to moderate, early AD was linked to an increased risk of developing childhood asthma [16]. These findings support the hypothesis of an atopic march in the general population that includes the progression of atopic diseases, consisting of asthma, AD, AR, and FA. Moreover, evidence shows strong epidemiological and pathophysiological association between AR and asthma in adults and children. In both adult and paediatric populations, the ARIA classifications of symptom duration (intermittent and persistent) and severity (mild, moderate, and severe) have been validated [6]. Furthermore, clinical characteristics and comorbidities play a significant role in the atopic march and the progression from AR to asthma is commonly documented in chronological order.

### 1.2. Treatment of Allergy

Paediatric immune responses are more flexible and may respond better to treatment; thus, interventions made early in life have a greater likelihood of changing the natural history of respiratory allergies [17]. A study revealed that 48% of children with persistent wheezing (the hallmark symptom of childhood asthma) and positive SPT had symptoms of AR but none of AR symptoms in children with early-transient wheezing [18]. Another study reported that prescribing inhaled fluticasone propionate to preschool children for wheezing had no effect on the natural history of asthma or wheeze later in childhood and that it did not prevent lung function decline or lower airway responsiveness [19]. There is substantial evidence of a relationship between AR and asthma, as well as a link between childhood AR and adult asthma [17]. The hypothesis is that AR may itself be an asthma risk factor. Therefore, asthma burden in later life may be reduced by more focused treatment of AR in childhood because the mechanism of AR usually underlies the clinical syndrome of asthma [17].

Current therapies can control allergic symptoms but are not a cure for allergic diseases. Current drugs, such as *β*2 agonist inhalers, antihistamines, and adrenaline, are used for allergy treatment and act on Th2-immune responses to inhibit the allergic disease [3]. Recent findings indicate that other immune responses are involved in allergic disease, including Th17-cells, Th1-type cytokines, and innate immune system, suggesting a promising therapeutic role of new agents that can block the action of these specific cytokines to improve the management of allergy and asthma. Specific immunotherapy to desensitise patients to allergens has been used for many years. However, risks of allergic reactions, including anaphylaxis, can occur as a consequence of this therapy and result in a life-threatening situation for the patient [3]. Other therapies being developed include targeting intrinsic structural defects, such as in the bronchial epithelium [5]. Nonetheless, the most effective way of reducing the overall burden of allergic disease is to implement early preventative strategies targeting allergic disease in children.

The administration of increasing amounts of specific allergens to which the patient has type I immediate hypersensitivity is known as allergen-specific immunotherapy (ASIT). AR, allergic asthma, and hymenoptera hypersensitivity are all treated with this disease-modifying therapy [20, 21]. Indications for ASIT include (1) insufficient symptom control despite pharmacotherapy and avoidance measures, (2) a goal to reduce AR and/or asthma morbidity, as well as the risk of anaphylaxis from future exposure, (3) when the patient is experiencing unfavourable pharmacotherapy side effects, and (4) when avoidance is not possible. Moreover, ASIT is cost effective compared with pharmacotherapy over a duration of time [20]. In AR and asthma therapy, the mode of ASIT administration is via the subcutaneous route by the physician or via the sublingual route and oral route by the patient [21]. Recent studies reported that immunotherapy appears to prevent the development of new allergy sensitizations and/or asthma in children with AR [17, 20]. Humoral, cellular, and tissue level changes occur with ASIT. These include large increases in anti-allergen IgG antibodies, a decrease in postseasonal rise of anti-allergen IgE antibodies, lower numbers of nasal mucosal mast cells and eosinophils, induction of Tregs, and inhibition of Th2 lymphocytes more than Th1 lymphocytes. A rise in IL-10 and TGF is shown as a result [20]. These recent studies suggest ASIT remains an important disease-modifying therapy in patients with allergic disease.

## 2. Early Detection of Allergy in Children

As allergic diseases usually occur in early life, early biomarkers of atopic susceptibility are required to target allergy and introduce preventive measures to high-risk infants. However, to date, there is no reliable general or specific allergy phenotype detection method that is easy and noninvasive for children. Most reported allergic phenotype detection methods are invasive, such as skin prick test (SPT), oral food challenge (OFC), and blood test, and many involve not readily accessible biological samples, such as cord blood (CB), maternal blood, or newborn vernix [22–27].

Early markers of atopic predisposition, such as cord serum IgE (CS-IgE) levels and maternal blood concentrations of IgE, have been used to target allergy-preventive measures in high-risk infants [24]. A high level of CS-IgE is thought to be a risk factor for subsequent allergies in children, and it can be used to predict atopic symptoms up to the age of 20. In Finnish populations, the combination of increased CS-IgE and a positive family history of allergy is strongly associated with subsequent atopic manifestations [24]. Nabavi et al. studied 181 Iranian neonates and their mothers showing that IgE maternal blood concentration was correlated with IgE concentrations in CB [25]. Further results showed that the presence of any kind of allergic disorder in the mother and elevated maternal blood IgE level was associated with CB IgE in the child [25].

In AD, SPT is the gold standard method for allergy diagnosis. SPT is invasive but is the main tool in allergy diagnostics. However, there is a mixed opinion regarding the clinical usefulness of SPT [27]. SPT enables the identification of people who are at risk for FA as well as the specific allergen that is causing the eczematous flare-up. Positive SPTs to foods, when performed by a nonspecialist, might lead to prolonged elimination diets, which can result in nutritional deficiencies, loss of tolerance to avoided foods, and increment of healthcare costs [22]. Therefore, there is a tremendous need for early, noninvasive biomarkers to identify individuals who are at risk of AD. Protein abundances in newborn vernix, such as polyubiquitin-C and calmodulin-like protein 5, show a strong negative correlation to the AD group [26]. Polyubiquitin-C and calmodulin-like protein 5 have the potential to replace SPT as a noninvasive allergy diagnosis in children and are promising candidates as biomarkers for identifying newborns predisposed to develop AD.

FA can be diagnosed using diagnostic decision levels and component-specific IgE. OFC remains the gold standard diagnostic for FA, but it is time consuming, expensive, and risky in terms of the child developing a severe allergic reaction during the test [27]. Nevertheless, OFC may also be an alternative way to reduce parental anxiety and improve education [23]. An ideal *in vitro* test, such as the clinical performance of microarray for specific IgE detection in children with challenge-proven/excluded cow's milk protein allergy (CMPA) or hen's egg (HE) allergy, could be a safer alternative to OFC [28]. D'Urbano et al. showed that in children with suspected CMPA or HE allergy, the microarray has a good ability to predict OFC results [28]. Furthermore, this approach decreases the number of OFCs performed and decreases the risk of a severe reaction; however, it is not cost effective [28]. Therefore, owing to severe reaction risk that may be caused by OFC, another cost-effective and noninvasive pretest is needed.

Anaphylaxis is diagnosed mostly based on clinical criteria and not on aberrant results from laboratory testing such as serum total tryptase levels. Anaphylaxis diagnosis is not fully excluded regardless of normal results in laboratory tests [29]. Asymptomatic sensitisation is common in the general population; thus, positive SPT or increased serum-specific IgE levels that test for potential triggering allergens confirm sensitisation but do not diagnose anaphylaxis [29]. Thus, identifying a biological test that is noninvasive, safe, and cost effective is urgently required.

Saliva is a biological sample that is easy to collect via a painless procedure. Furthermore, saliva is the best approach for biomarker measurement as it is easily accessible, noninvasive, and an easy bedside screening for real-time measurement of the ongoing human physiological system [30–32]. All of these benefits highlight saliva as a very promising diagnostic candidate for detection and monitoring of disease biomarkers, especially in infants, toddlers, children, and anxious or uncooperative patients [33]. However, to date, saliva is not considered a medically acceptable biomarker. Interestingly, recent studies of saliva suggest that it can be used for the detection of head and neck carcinoma, breast and gastric cancers, salivary gland disease, Sjögren syndrome, systemic sclerosis, dental and gingival pathology, preeclampsia, and psychiatric and neurological diseases [30, 34–38]. Protein biomarkers have the advantages as modifiable influencing factors rather than genetic and epigenetic factors that are mostly nonmodifiable factors for allergic disease susceptibility in childhood. Saliva has great potential to replace serum as a biological fluid biomarker in diagnosing clinical allergy, especially in infants, toddlers, children, anxious, and uncooperative patients [33]. Moreover, saliva is an easily accessible, noninvasive, real-time measurement of the ongoing human physiological system and consists of an abundance of biomarkers, such as genetic material and proteins [30–32].

This review considers whether the saliva could be suitable biological samples for early detection of allergic risk. Such tools may be used as justification for targeted interventions in early childhood for disease prevention and assist in reducing morbidity and mortality caused by childhood allergy.

## 3. Biological Marker (Biomarker)

### 3.1. Criteria for a Biological Marker (Biomarker)

A biological marker (biomarker) is a characteristic that is objectively measured and evaluated as an indicator of normal biologic processes, pathogenic processes, or pharmaceutical responses to therapeutic intervention, according to the National Institutes of Health [39, 40]. Any biomolecule or a specific characteristic, feature, or indicator of a change in any biological structure and function that can objectively measure the state of a living organism is referred to as a biomarker [41].

The criteria for biomarkers include

1. A significant oxidative modification product that may be directly linked to the onset of illness
2. A stable product that is resistant to artefact induction is difficult to lose and does not change throughout storage
3. Representative of the balance between oxidative damage generation and clearance
4. Verified by an analytical assay that is specific, sensitive, reproducible, and robust
5. Free of confounding and interference variables from dietary consumption
6. Accessible in a target tissue or a valid surrogate tissue such as a leukocyte
7. Detectable and quantifiable within the limits of detection of a reliable analytical procedure [40, 42]

### 3.2. Criteria for Potential Salivary Protein Biomarkers for Allergy

Therefore, to choose salivary protein targets for this current study, specific criteria for selection were defined. The criteria include

(1) Exclude proteins that are
  - Abundantly expressed in saliva
  - Associated with other pathologies
  - Altered in an inflammatory response in a nonspecific manner
  - That change with age and sex
(2) Include proteins that are
  - Acknowledged to be associated with the disease of interest
  - Not commonly observed in saliva

In addition, a confounding factor that should be considered includes proteins that are produced by the salivary glands versus proteins that enter saliva by diffusion from the circulation.

## 4. Saliva Samples

### 4.1. Types of Saliva Samples

There are two types of salivation, unstimulated and stimulated salivation. Unstimulated salivation is watery saliva produced by the salivary gland at rest, reflects the basal salivary flow rate, and is stimulated by parasympathetic innervation. Unstimulated salivation occurs for about 14 hours a day, and 90% of this saliva is produced by major salivary glands [43]. On the contrary, stimulated salivation represents the thicker secretion during food intake and is stimulated by sympathetic innervation. This saliva is present in our mouths for up to two hours and contains more salivary protein in the afternoon than in the morning; its concentration follows this diurnal pattern [43].

Stimulated salivation is preferable especially in children, in which the children can easily chew on the swab to stimulate saliva production.

### 4.2. Saliva Sample Collection Method

**Figure 1:**
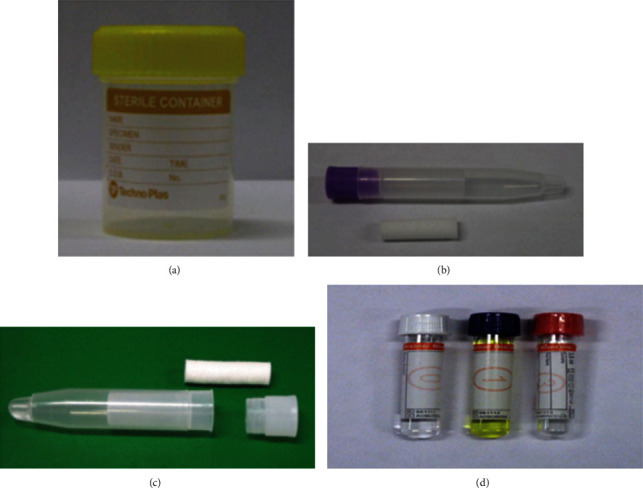
Commercially available saliva collection devices [44]. (a) Drool collected in a sterile specimen container. (b) SalivaBio Oral Swab (SOS) (Salimetrics, Carlsbad, California, the United States). (c) Salivette: cotton and synthetic swab (Sarstedt, Thermo Fisher Scientific, California, United States). (d) GBO Saliva Collection System (Greiner Bio-One, Kremsmünster, Austria).

Four commercially available saliva collection devices are described in Figure 1 and include

1. Unstimulated saliva: drool and SalivaBio Oral Swab (SOS) (Salimetrics, Carlsbad, California, the United States)
2. Mechanically stimulated saliva: Salivette with a cotton swab (Sarstedt, Thermo Fisher Scientific, California, United States)
3. Mechanically stimulated saliva: Salivette with a synthetic swab (Sarstedt, Thermo Fisher Scientific, California, United States)
4. Acid stimulation saliva: GBO Saliva Collection System (Greiner Bio-One, Kremsmünster, Austria)

There are variations of salivary protein composition and salivary flow rate depending on the methods used in saliva sample collection [44]. The standard drool method (Figure 1(a)) had significantly higher salivary protein concentration as compared with the GBO Saliva Collection System method (Figure 1(d)). Furthermore, when compared to mechanically stimulated methods, salivary flow rates were significantly lower in unstimulated saliva, which includes drool (Figure 1(a)) and SalivaBio Oral Swab (SOS) (Figure 1(b)). These findings revealed significantly relevant differences in analyte levels and the salivary flow rates are determined by the saliva collection method [44].

However, based on the current study population having children between 6 months to 5 years old, mechanically stimulated saliva using Salivette with a cotton swab (Sarstedt, Thermo Fisher Scientific, California, the United States) (Figure 1(c)) was the most suitable saliva collection device to be used in children. This method was chosen as it was an easy, noninvasive, and painless procedure [32]. Unstimulated saliva using drool or SOS and acid stimulation saliva using the GBO Saliva Collection System is not feasible and can be extremely difficult to perform on these children.

### 4.3. Children Saliva Sample Collection Protocol Using Salivette

There are two methods to collect the children saliva, depending on the child's age, using Salivettes with a cotton swab (Sarstedt, Thermo Fisher Scientific, California, United States) or the standard drool method. These two methods were chosen and conducted according to the flexibility and cooperation of the child during the saliva sample collection procedure. The saliva sample collection procedure was conducted during a fixed time between 8.30 in the morning to 12 noon to enable the researcher to minimize the baseline variations, thus reducing the diurnal variations of salivary proteins.

**Figure 2:**
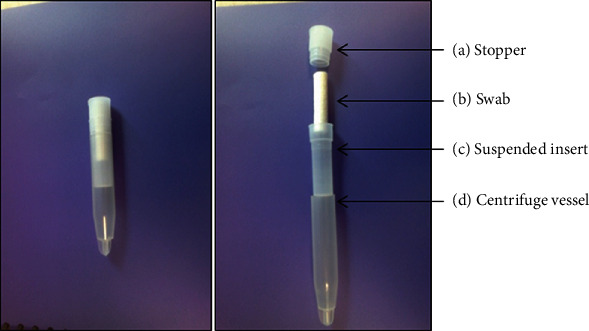
Salivette's parts. (a) Stopper. (b) Swab. (c) Suspended insert. (d) Centrifuge vessel.

If the child is less than 3 years old, the drooling saliva was collected from the child's mouth using the tube or swab. First, the Salivette was held at the rim of the suspended insert (Figure 2(c)) and the stopper is parted (Figure 2(a)) by slightly pushing it to the side. Then, the swab (Figure 2(b)) is removed from the Salivette and the tube is placed at the tip of the child's mouth to collect the drooling saliva in younger children (Figure 2).

**Figure 3:**
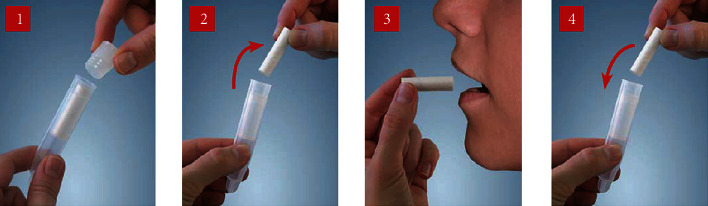
Steps of Salivette collection method. (1) The Salivette is held at the rim of the suspended insert, and the stopper is parted by slightly pushing it to the side. (2) The swab is removed from the Salivette. (3) Let the child chew the swab in his or her mouth for 1 minute, or the tube is placed at the tip of the child's mouth to collect the drooling saliva. (4) The swab is put back to the suspended insert, and the Salivette is closed firmly with the stopper.

Older children were asked to spit inside the tube, or saliva was collected from the child's mouth by inserting the cotton swab and allowing them to hold or chew it in their mouth for 1 minute (Figure 3). Approximately 1 ml of saliva volume was collected with both methods.

After completion of the saliva collection, the Salivette was centrifuged at 3500 rpm for 30 minutes at 4°C. The centrifugation was performed within 4–6 hours after the saliva collection to avoid further protein degradation. A 10 *μ*l aliquot of protease inhibitor cocktail (PIC) (*v*/*v* 1 : 100), Sigma Protease Inhibitor Cocktail (Sigma-Aldrich, St. Louis, Missouri, the United States), was added to the Salivette before saliva collection to inhibit protein degradation. The Salivette and saliva were kept on ice (0 to −4°C) during the whole collection procedure as PIC will degrade if kept at a temperature higher than 4°C. After centrifugation, the solution of saliva and PIC was mixed and aliquoted to several cryotubes containing at least 200 *μ*l of saliva and stored at −80°C. Approximately 200 *μ*l of saliva was required for the proteomic analysis; therefore, only 1 tube was thawed at one time and this minimized the protein degradation effect on thawing and refreezing of the saliva samples.

### 4.4. Saliva Sample Handling

Sample handling imposes a real challenge to saliva analysis. Saliva samples need to be collected and stored under conditions of minimal proteolysis, deglycosylation, and dephosphorylation to minimize protein degradation [45]. Protein degradation may be caused by several factors: A variety of factors can cause protein degradation including bacterial proteases that may present in saliva, high temperature, pH conditions, and the freezing and thawing cycle. [46]. The recommended protocol to prevent any degradation is that sample processing should be performed on ice (0 to 4°C), and PIC is added immediately, centrifuged to remove insoluble material, and stored in −20 or −80°C [47]. However, without PIC, short-term storage (less than 24 hours) of freshly collected samples on ice is effective in preventing protein degradation without compromising the chemistry of the proteome [46]. Furthermore, minimizing the time elapsing between sample collection and analysis will potentially reduce the risk of protein degradation. All these influencing factors must be carefully considered to prevent protein degradation as minimal as possible.

## 5. Salivary Protein Confounding Factors and Childhood Allergy

**Figure 4:**
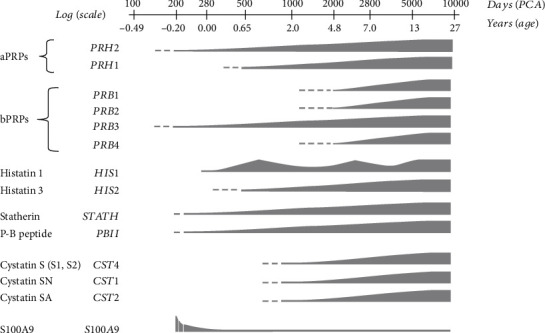
Scheme describing the approximate time course of different salivary proteins and peptides [48]. A function of age is the sum of extracted ion chromatogram (XIC) peak regions of all members belonging to the same family of proteins/peptides that are grouped based on the locus. A logarithmic scale was used in the abscissa axis to better highlight major changes in preterm newborns during the first months of life, which corresponds to the last months of fetal development, and those that occur immediately after the normal term of delivery and in the first years of life. Dashed lines indicate the age range in which the proteins were detected in at least one baby. The highest limit of this range corresponds to the age at which all saliva of the babies, even in small amounts, displayed the protein in the chromatographic profile.

In the process of identifying potential salivary biomarkers for allergy, meticulous attention should be given to some salivary proteins that are dependent on age, sex, or state of disease, especially caries lesions or periodontal disease [48, 49]. Messana et al. investigated that the human salivary proteome was studied in a large sample of subjects of various ages, ranging from preterm newborns of 180 days of postconceptional age to 17 year olds. The study defined the appearance and level changes in proteins typically found in adult saliva from the last months of fetal development to adulthood [48]. Evidence suggested that individual salivary proteome diversity is particularly significant in paediatric age, especially in preterm newborns, such as S100 proteins (Figure 4) [48]. This was founded on the principle that proteome variability, which occurs as a result of physiological changes over time, has a significant impact. Exploration of proteomic temporal changes is termed “chrono-proteomics” [48]. In relation to sex specificity, a study evaluated interindividual biochemical variation in a population of 268 systemically healthy young students [49]. Findings revealed that female participants had lower salivary pH, buffering capacity, protein content, MUC5B, secretory IgA, and chitinase activity than male subjects, whereas male subjects had higher MUC7 and lysozyme activity [49]. The findings demonstrate that essential salivary biochemical variables have distinctive distributions and interrelationships in a systemically healthy young adult population, emphasising significant gender variations in salivary biochemistry [49].

## 6. Saliva as Potential Diagnostic Fluid in Childhood Allergy

Previously, there has been controversy related to the use of saliva as a diagnostic fluid due to the low concentration of analytes in saliva compared to blood. Nevertheless, technological advances, increased sensitivity of molecular methods, and nanotechnologies, such as mass spectrometry (MS); have reduced these limitations [50]. The advantages of using saliva as a diagnostic fluid are listed in Table 1 [33].

Approximately 2700 salivary proteins have been identified, and this number is expected to increase dramatically in the future due to current advances in detection methods for biomarkers in saliva [30, 51]. Saliva proteins identified to date have been categorised according to their functions (Table 2). The main function is immunity that contributes 21% of all known salivary proteins [34]. This suggests that this particular fluid is a suitable biological fluid for identifying a biomarker of allergic risk.

Most diseases, such as cancer and allergy, are likely to be treated efficiently if the disease can be diagnosed early. For example, ovarian cancer, the fifth most frequent malignancy and the leading cause of death in females, has a five-year survival rate of 25% when detected at stage 4, compared to 93% when diagnosed at stage 1 [52]. Saliva has been used widely to detect a variety of diseases using proteomic approaches, including head and neck carcinoma (oral cavity, oropharynx, larynx, and salivary glands), breast and gastric cancers, salivary gland function and disease, Sjögren syndrome, systemic sclerosis, dental and gingival pathology, preeclampsia, and psychiatric and neurological diseases [30, 34–38] (Tables 3 and 4). Childhood susceptibility to allergy is most likely possible to be detected in saliva using a proteomic approach to identify novel proteins that vary with allergy.

## 7. The Role of Saliva in Early-Life Allergy Detection

Saliva is a human body fluid with complicated constituents and various biological functions [31, 34, 53]. The concentrations of analytes in saliva are 1000-fold lower than those in human serum [53]. Like the serum, saliva contains hormones, amino acids, electrolytes, immunoglobulin(Ig), antibodies, growth factors, enzymes, microbes, and their products. The majority of these constituents enter saliva via passive diffusion, active transport, or extracellular ultrafiltration through blood barriers of capillary walls [53]. Furthermore, expectorated saliva contains a significant amount of total gingival crevicular fluid (GCF) from periodontal pockets throughout the mouth [54]. GCF is an inflammatory serum exudate produced by periodontal tissue inflammation that originates from the blood vessels in the gingival connective tissue [55]. It contains biological molecular markers accumulated from the systemic and surrounding circulation [38]. The GCF is composed of a complex combination of molecules from the blood, the host tissues, and the subgingival biofilm, including leucocytes, proteins, enzymes, tissue breakdown products, inflammatory mediators, and cytokines produced locally in reaction to the bacterial biofilm [55]. GCF is a good source of biochemical disease markers because it can accurately reflect the ongoing response of periodontal cells and tissues. Hence, saliva, which also contains GCF, is an important bodily fluid that reflects the physiological and pathological function of the human body [31].

Saliva's essential functions in the oral cavity are lubrication and binding, followed by solubilization of dry food, oral hygiene, starch digestion initiation, and immunity [36]. Lubrication aids in lubricating the hard and soft oral surfaces and is vital for speaking, mastication, and swallowing by providing a lubricious layer that contains mucins, proline-rich proteins, and water [53]. Saliva aids in bolus formation by moistening food, which protects the oral mucosa from mechanical damage. In addition, saliva aids in the preliminary digestion of food by containing salivary alpha-amylase, which breaks down carbohydrates into sugars, while salivary lipase initiates fat digestion [53]. It also facilitates taste perception and digestion of carbohydrates by salivary alpha-amylase. This process allows soluble food-derived molecules to enter the gustative papillae and buffer the acidity of the food with the bicarbonates [50].

Saliva also contains lysozyme, an enzyme that lyses bacteria and inhibits oral microbial populations from overgrowing [53]. The antibacterial and antiviral properties, as well as its maintenance of tooth and mucosal integrity, are mostly due to salivary mucins, which bind to bacteria and prevent bacterial adhesion to tooth enamel. Saliva proteins are identified according to their functions. Interestingly, the main known function of salivary proteins is immunity, with 21% of salivary proteins being involved in immune function [34]. Therefore, saliva is a promising biological sample that may contain biomarkers of allergic disease risk.

Proteomic technologies, which combine modern instrumentation and enhanced analytical procedures, are widely used in clinical applications involving biomarkers. Due to its high sensitivity and precision for mass measurement, mass spectrometry- (MS-) based approaches for salivary biomarker identification have become one of the core technologies for proteomics in the last decade [56]. These include a variety of MS techniques, such as two-dimensional gel electrophoresis–mass spectrometry (2-DE/MS), liquid chromatography-tandem mass spectrometry (LC-MS/MS), matrix-assisted laser desorption/ionisation time-of-flight mass spectrometry (MALDI-TOF/MS), and surface-enhanced laser desorption/ionisation time-of-flight mass spectrometry (SELDI-TOF/MS), which have been used to identify biomarkers in saliva on a large scale, but not in relation to allergic diseases (Table 5) [56]. Although MS has been used extensively, most studies have moderate sample sizes (less than 45) as MS is an expensive method. Therefore, validation methods used, such as ELISA and Western blot, with larger sample sizes have been used to confirm MS findings (Table 5).

## 8. Conclusions

Saliva sampling is a noninvasive and stress-free alternative to blood collection; thus, there is no discomfort or pain associated with blood venepuncture. It is a readily accessible secretion that is generally recognised as a possible clinical diagnostic medium [56]. Moreover, as compared with blood, saliva contains fewer proteins which reduce the possibility of nonspecific interference and hydrostatic interactions [40]. Protein concentrations in the blood can range from a few seconds to several months or longer, with protein half-lives ranging from a few seconds to several months or longer. Interestingly, the composition of saliva is less complex and variable than serum; therefore, it should accurately reflect the current condition of the body at any given time [40]. Furthermore, as compared to blood, other advantages include easy and multiple sampling opportunities, less need for sample preprocessing and hence cost effectiveness, and minimal risk of contracting infectious organisms, and it is also an ideal biofluid for collecting specimens from patients in developing countries [56, 57]. Thus, saliva has the promising potential to replace blood as the gold standard in diagnosing allergic diseases.

## Figures and Tables

**Table 1:** Advantages of salivary testing for diagnosis [33].

Advantages of salivary testing for diagnosis
Noninvasive, simple to use, and low cost
Safer to conduct than serum sampling (needles are not required)
Diagnostic values in real time
No need for trained medical professionals
Obtaining many samples is simple
At-home collection and screening are possible
Cross contamination risks are minimal
More cost-effective sampling, shipping, and storage compared to serum
Requires less manipulation during diagnostic procedures compared to serum
Screening assays are commercially available

**Table 2:** Saliva proteins are identified according to their functions. The table is adapted from [34].

Salivary protein percentage (%)	Salivary functions
28.7	Uncertain function
21.0	Immunity
15.4	Unknown function
9.7	Signal transduction
7.1	Cytoskeleton and endomembrane
5.2	Metabolism
4.8	Cell mobility and secretion
4.2	Cell multiplication and cell cycle
2.3	Transcription and ribosomes
1.6	Protein replication and reparation

**Table 3:** Salivary proteins that are involved in oncological pathologies [30].

Author, year	Proteins involved	Proteins not involved	Site of tumor
Nakashima et al., 2006	(i) Maspin (ii) Stathmin	—	Salivary gland

Contucci et al., 2005	—	(i) Statherin	Salivary gland

An et al., 2004	(i) Transketolase (ii) Dim1p (iii) v-Ha-ras oncogene (iv) Type I collagen pro alpha (v) Tumor necrosis factor (ligand) superfamily member 4 (vi) Pirin	—	Salivary gland with metastasis

Oshiro et al., 2007	(i) Alpha-1-B-glycoprotein (ii) Complement factor B proteins	(i) Cystatin S (ii) Parotid secretory factor (iii) Poly-4-hydrolase beta-subunit proteins	Head and neck

Dowling et al., 2008	(i) Beta fibrin (ii) S100 calcium-binding protein (iii) Transferrin (iv) IG heavy chain constant region *γ* (v) Cofilin-1 (vi) Transthyretin	—	Head and neck

Mizukawa et al., 2001	(i) Alpha-defensins (ii) Beta-defensins	—	Oral cavity

Contucci et al., 2005	(i) Statherins	—	Oral cavity

Wong, 2006	(i) Interleukin-8 (IL-8) (ii) Thioredoxin	—	Oral cavity

Pickering et al., 2007	(i) Endothelins	—	Oral cavity

Streckfus et al., 2000	(i) c-erbB-2 protein (ii) CA15-3	—	Breast

Tabak, 2001	(i) 15-3 cancer antigen	—	Breast

**Table 4:** Salivary proteins that are involved in dental pathologies [30].

Author, year	Proteins	Related pathology
Dowd, 1999; Van Nieuw et al., 2004	(i) Mucins (ii) Proline-rich glycoprotein (iii) Statherin	Dental caries

Vitorino et al., 2006	(i) Proline-rich proteins (ii) Lipocalin (iii) Cystatins (iv) Amylase (v) Immunoglobulin a (vi) Lactoferrin	Dental caries

Rudney et al., 2009	(i) Statherin (ii) Truncated cystatin S	Dental caries and other diseases

Nishida et al., 2006	(i) IL-1 beta (ii) Albumin (iii) Aspartate aminotransferase	Periodontitis

Kibayashi et al., 2007	(i) Prostaglandin E(2) (ii) Lactoferrin (iii) Albumin (iv) Aspartate aminotransferase (v) Lactate dehydrogenase (vi) Alkaline phosphatase	Periodontitis

Fábián et al., 2007; 2008	(i) Immunoglobulin (ii) Molecular chaperone hsp70 (iii) Cystatin S (iv) Salivary amylase (v) Calprotectin (vi) Histatins (vii) Lysozyme (viii) Lactoferrin (ix) Defensins (x) Peroxidases (xi) Proline-rich proteins (xii) Mucins	Periodontitis

Ito et al., 2008	(i) Cystatins (ii) Lysozyme	Periodontitis

**Table 5:** Summary of MS-based methods used for salivary biomarker identification. The table is adapted from [56].

Disease	Saliva	Stimulation	Proteomics approach	Biomarkers	Verification methods	References	Sample size
Breast cancer	Whole	Stimulated	SELDI-TOF/MS	(i) c-erbB-2	(i) ELISA (ii) Western blot	Streckfus et al., 2006	Control—3 Disease—3

Caries	Whole	Unstimulated	2-DE/MS	(i) Statherin 5 (ii) Cystatin	(i) Western blot	Rudney, et al., 2009	Control—18 Disease—23

Gastric cancer	Whole	Unstimulated	MALDI-TOF/MS	(i) 1472.78 Da (ii) 2936.49 Da (iii) 6556.81 Da (iv) 7081.17 Da	—	Wu, 2009	Control—18 Disease—23

Graft versus host disease	SM/SL	Stimulated	SELDI-TOF/MS MALDI-TOF/MS	(i) Lactoferrin (ii) SLPI (iii) IgA (iv) b2-microglobulin	(i) ELISA	Imanguli et al., 2007	Control—0 Disease—41

HNSC	Whole	Stimulated	LC–MS/MS	(i) Complement factor B	(i) Western blot	Ohshiro et al., 2007	Control—5 Disease—3

Oral lichen planus	Whole	Unstimulated	2-DE MALDI-TOF/MS	(i) Urinary prokallikrein (ii) PLUNC	—	Yang et al.,2006	Control—6 Disease—6

OSCC	Whole	Unstimulated	2-DE/MS LC–MS/MS	(i) M2BP (ii) Catalase (iii) Profiling1 (iv) CD59 (v) MRP14	(i) ELISA (ii) Western blot	Hu et al., 2008	Control—64 Disease—64

Pulmonary disease	Whole	Unstimulated	2-DE/MS	(i) Lipocalin (ii) Apolipoprotein A1	—	Nicholas et al., 2010	Control—20 Disease—25

Type 1 diabetes	Whole	Stimulated	2-DE MALDI-TOF/MS	(i) *α*-amylase (ii) Cystatin (iii) PIP	—	Hirtz et al., 2006	Control—8 Disease—8

Type 2 diabetes	Whole	Unstimulated	LC–MS/MS	(i) A1AT (ii) *α*-2-macroglobulin (iii) Cystatin C (iv) Transthyretin	(i) ELISA	Rao et al., 2009	Control—10 Disease—30

SM represents submandibular; SL represents sublingual; HNSC represents head and neck squamous carcinoma; OSCC represents oral squamous cell carcinoma; SS represents Sjögren's syndrome.
